# Deepening Our Understanding of the Factors Affecting Landscape of Myeloproliferative Neoplasms: What Do We Know about Them?

**DOI:** 10.3390/cancers15041348

**Published:** 2023-02-20

**Authors:** María Luz Morales, Francisca Ferrer-Marín

**Affiliations:** 1 Hematology and Medical Oncology Department, Hospital Universitario Morales-Meseguer, Centro Regional de Hemodonación, IMIB-Pascual Parrilla, 30120 Murcia, Spain; 2 CIBERER CB15/00055, 28029 Murcia, Spain; 3 Grade of Medicine, Faculty of Health Sciences, Catholic University of Murcia (UCAM), 30170 Murcia, Spain

**Keywords:** myeloproliferative neoplasms, transcriptomics, proteomics, microbiota, metabolism

## Abstract

**Simple Summary:**

Since the discovery of the role of the three driver mutations (*JAK2*, *CALR* and *MPL*) and the constitutive activation of the JAK/STAT pathway in myeloproliferative neoplasms, the disease has been wrongly considered to be molecularly elucidated, and efforts have mainly focused on the implementation of JAK inhibitors in the clinical setting. This review aims to bring together some of the most relevant studies addressing factors other than genetic alterations that may also play a role in the origin, evolution and progression of the disease and could, therefore, be the target of new therapies as well.

**Abstract:**

Myeloproliferative neoplasms (MPNs) arise from the uncontrolled proliferation of hematopoietic stem and progenitor cells in bone marrow. As with all tumors, the development of MPNs is a consequence of alterations in malignant cells and their interaction with other extrinsic factors that support and promote tumor progression. Since the discovery of driver mutations, much work has focused on studying and reviewing the genomic features of the disease but has neglected to delve into the important role that many other mechanisms may play. This review discusses the genetic component of MPNs but focuses mainly on some of the most relevant work investigating other non-genetic factors that may be crucial for the disease. The studies summarized here address MPN cell-intrinsic or -extrinsic factors and the interaction between them through transcriptomic, proteomic and microbiota studies, among others.

## 1. Introduction

The 5th edition of the World Health Organization (WHO) classification [1] of hematolymphoid tumors, as well as the International Consensus Classification of Myeloid Neoplasms [2], recognized eight subtypes of MPN disease. This review is focused on Philadelphia-negative classical chronic myeloproliferative neoplasms, hereafter referred to as MPNs, which comprise polycythemia vera (PV), essential thrombocythemia (ET), primary myelofibrosis (PMF) and prefibrotic myelofibrosis (MF) [1,2]. They are *BCR*:*ABL*- negative hematological malignancies characterized by the clonal proliferation of hematopoietic stem and progenitor cells (HSPC), myeloid precursors and mature myeloid cells, with or without bone marrow (BM) fibrosis [3].

MPN patients show clinical features (i.e., splenomegaly, thrombotic complications, progression to myelodysplasia or acute leukemia) and a common molecular basis [4]. The main mutations in MPNs occur in *JAK2*, *CALR* or *MPL* and lead to the constitutive activation of JAK/STAT signaling (driver events). The genetic aberrations that perturb JAK/STAT signaling provoke the overproduction of mature and functional blood cells from hematopoietic stem cells (HSC) in response to growth factors and cytokines [5]. In over half of patients with MPNs, these diseases are associated with acquired mutations in other myeloid genes, which are involved in DNA methylation, chromatin modification, signaling and mRNA splicing [5]. Importantly, these additional mutations are frequent in advanced stages of the disease (MF) compared to earlier chronic stages (PV and ET), and some are known to correlate with a poorer prognosis risk [5,6].

Despite the fact that MPN patients have a shorter survival than the general population of the same age and sex, which is much lower in PMF (5–7 years) than in PV or ET (14 and 20 years, respectively) [7], the treatment of these neoplasms is aimed at symptom control and the prevention of vascular complications. Thus, among patients with PV and ET, the most prevalent forms, survival is mainly determined by thrombotic complications and progression to secondary MF, or, less frequently, to acute myeloid leukemia (AML) [8]. In spite of this risk of progression to more advanced stages, current risk stratification in PV and ET is exclusively designed to estimate the likelihood of recurrent thrombosis. In relation to MF, none of the current available treatments, except hematopoietic stem cell transplantation (HSCT), has a curative potential or is able to modify the natural history of the disease [9]. In fact, although almost 95% of PV and 50–60% of ET and PMF patients harbor *JAK2* mutations [5], clinical experience suggests that targeting JAK2 may not be sufficient to eliminate malignant mutated cells [10].

Although the MPN genome is well known in terms of spectrum, frequency and the order of acquisition of the somatic mutations [5,11], the lack of efficacy of current treatment options, even if they are targeted therapies, suggests the implication of other important factors in the pathophysiology of MPNs. Certainly, although MPNs could be considered a genetically simple disease, they show widely heterogeneous phenotypes, displaying the interplay between genetic and non-genetic factors.

In this review, we will summarize several of the most relevant studies exploring different intrinsic and extrinsic factors, with some of them described as hallmarks of cancer. A better understanding of the factors that break into MPN development and progression is necessary for outcome improvement.

## 2. Intrinsic Factors

The clonal evolution of MPNs is determined by the interaction of several factors: those related to the malignant cell and its progeny (genomics, transcriptomic and proteomic alterations) and those cell-extrinsic mediators and factors, such as microbiota and environmental exposures.

In this section, we will focus on cell-intrinsic mechanisms that have been evaluated and are somewhat related to MPNs.

### 2.1. Genomic Studies

The molecular pathogenesis of *BCR::ABL*-negative myeloproliferative neoplasms was unascertained until 2005, when the presence of an activating point mutation in the *JAK2* gene was first described [12,13,14,15]. Thereafter, other driver mutations events have been elucidated, such as the presence of mutations in *MPL* (which encodes the thrombopoietin receptor, TPOR) [16] or *CALR* [17,18]. These three genes are considered disease drivers as, when mutated, they could provoke MPN development in murine models [19].

#### 2.1.1. Classical Driver Mutations

The canonical and most common *JAK2* mutation is a single nucleotide change at codon 617, located in exon 14, resulting in the substitution of a valine for phenylalanine (V617F). This amino acid shift in the JH2 pseudokinase domain prevents the autoregulatory inhibition of JH1, resulting in the constitutive phosphorylation and activation of the Janus kinase [13]. Roughly one third of PV patients who are *JAK2*V617F-negative harbor typically insertions or deletions in *JAK2* exon 12 [20]. Both mutations lead to a gain-of-function of the kinase, the consequent activation of its downstream targets and deregulation of JAK/STAT signaling [5]. Moreover, the *JAK2*V617F allele burden has been associated with the MPN subtype, progression to secondary MF and an increased thrombotic risk [6,21,22].

The conventional *MPL* mutations take place in W515, located in exon 10, resulting in amino acid substitutions [23], with W515L/K being the most common changes [24]. Serine 505 could be also mutated, though less frequently [25]. All these mutations prevent the inactive conformation of the TPOR receptor, leading to its cytokine-independent activation and the constitutive JAK/STAT pathway activation [26].

*CALR* mutations present in MPN patients are insertions and deletions located at the exon 9, generating a novel C-terminal peptide sequence [17]. Almost 85% of *CALR*-mutated patients harbor a deletion of 52 bp (type I, *CALR^del52^*) or an insertion of 5 bp (type II, *CALR^ins5^*) [26] and drive JAK/STAT pathway activation through TPOR activation [11].

Although driver mutations were classically considered as mutually exclusive, some studies have identified double mutated patients (i.e., *JAK2*V617F and *CALR*, *JAK2*V617F and *MPL*, *CALR* and *MPL*, and *JAK2*V617F and *JAK2* exon 12 mutations) [27], arising from different HSC clones. Recent work suggests that *CALR* mutations tend to occur later in life than *JAK2*V617F, which would explain the higher proliferative advantage of the *CALR* malignant clone compared to *JAK2*V617F [28]. 

Finally, in recent years, there has been a growing body of evidence suggesting, in some cases, an in utero or postnatal acquisition of MPN-driver mutations [29]. In line with this, it has been estimated that the *JAK2*V617F mutation could occur in a single HSC several decades before MPN diagnosis [30,31]. A better understanding of the evolutionary dynamics of MPNs, in addition to the long disease latency, might open up opportunities for early intervention.

#### 2.1.2. Triple Negative Patients

Considering that these three mutations have remained the main drivers of the disease for years, patients who exhibit histological and phenotypic MPN features but lack either of these mutations have classically been known as “triple negative” (TN) patients. Around 10–15% of ET and PMF patients are triple negative [32]. The investigation of these TN patients with next generation sequencing (NGS) approaches showed that many other mutations may affect MPN patients, especially those classified as TN [5,11]. Furthermore, delving deeper into this particular group of patients, sequencing studies have revealed that TN ET patients showed the dysregulation of genes involved in MAPK, tumor necrosis factor and NF-κB pathways, leading to activation of the JAK/STAT pathway [33].

#### 2.1.3. Mutations in Other Myeloid Genes

Some relevant patient cohort studies have sequenced 104 genes in 197 MPN patients and followed clonal evolution relating the number of somatic mutations identified with reduced overall survival and an increased risk of AML transformation [34]. Larger studies sequenced coding exons from 69 myeloid cancer genes in 1887 patients [6] and developed a new personalized prognosis classification for MPNs. Forty-five percent of patients from this cohort showed mutations in *JAK2*, *CALR* or *MPL*, whereas the remaining 55% were classified as TN MPNs. Authors defined eight genomic subgroups with different clinical phenotypes creating a prognostic model tool.

In general, besides *JAK2*, *CALR* or *MPL* mutations, many others contribute to the MPN phenotype. As previously mentioned, the presence of additional mutations is usually more common in advanced stages of the disease (MF) than in earlier chronic stages (PV and ET) [5,6]. The most affected genes are involved in DNA methylation, mRNA splicing and signaling with mutations in *TET2*, *DNMT3A* and *ASXL1* found in almost half of patients [1] but also *EZH2* [35], *CBL*, *PPM1D*, *SF3B1*, *NFE2*, *TP53*, *SRSF2* and *U2AF1* [6] as the most frequent ones. 

##### Mutations in Genes Involved in DNA Methylation

Mutations in *DNMT3A*, *TET2* and *ASXL1* (DTA) genes confer a selective advantage that may lead to an expanded clone of cells, termed clonal hematopoiesis, which is an age-related pre-malignant condition. The mutations associated with clonal hematopoiesis of indeterminate potential (CHIP) increase cardiovascular risk and associated mortality [36,37]. The study of additional CHIP mutations and their association with increased cardiovascular risk has attracted a great deal of research interest in MPNs, and some authors suggest that DTA mutations, particularly *TET2* mutations, may be an independent risk factor for thrombosis in PV [38]. In contrast, the presence of mutations in *ASXL1*, *RUNX1* or *EZH2* seems to play a salutary effect on the risk of arterial thrombosis, particularly in ET patients [39].

Although in previous studies on MPNs, mutations in genes involved in DNA methylation had not been related to blast phase progression [40], it is of relevance that they have been described as a risk factor for relapse after treatment for AML [41]. Indeed, more recent studies in a large cohort of Japanese PV and ET patients identified *ASXL1* mutations as a risk factor for leukemic/myelofibrotic transformation [42].

Furthermore, *ASXL1* [43] or *EZH2* mutations [44] by themselves have been related to poorer survival in PMF patients. In fact, when both mutations coexist, MPN patients present a higher risk of secondary MF transformation and poorer survival [45]. Therefore, mutations in these genes have been included as high risk mutations (HRMs) in the Mutation-Enhanced International Prognostic Score System for Transplantation-Age Patients With Primary Myelofibrosis (MIPSS70 score) [46].

##### Mutations in Splicing Components

Despite being particularly more common in myelodysplastic syndrome (MDS) patients [47], mutations in the splicing factors *SF3B1*, *SRSF2* and *U2AF1*, between others, represent the second most frequently mutated gene category in MPNs [48], especially in PMF, and have been linked to a higher risk of AML transformation as well as lower overall survival [45]. Notably, whereas *SF3B1* is the most frequent mutated splicing factor in MPNs [49], different outcomes could be observed for each MPN entity. When mutated, it is associated with lower overall survival in ET [50] and secondary MF (particularly post-ET patients) [51] but not in PV [50] or PMF patients [52]. Specially, it is also related to lower myelofibrosis-free survival [50]. Conversely, *SRSF2* mutations in all MPN patients predict adverse outcomes [50,53], and they are considered HRMs in the MIPSS70 score [46].

##### Mutations in Genes Involved in DNA Repair and Other Signaling Pathways

*TP53* mutations are also particularly very common in MDS patients [54]. Although their presence in MPNs is lower, patients harboring *TP53* mutations have a dismal prognosis and a higher risk of AML transformation [6,34,50], especially when accompanied by a proinflammatory microenvironment [55]. Not only *TP53*, but also mutations in components of other signaling pathways, such as *PPM1D* or *NRAS*, have been related to leukemic transformation and early death [35].

Altogether, in the last decade, genomic studies have identified multiple recurrent somatic mutations in MPNs. It is, therefore, intriguing how the same genetic background may have completely different disease courses, with completely different phenotypes, responses to treatment and risks of thrombotic events or leukemic progression. This fact underlines the importance of evaluating MPN biology at other levels beyond the mutational study in order to be able to anticipate the worsening of patients or administer the best therapeutic options in each case in a precise manner.

### 2.2. Transcriptomic Analyses

**Figure 1:**
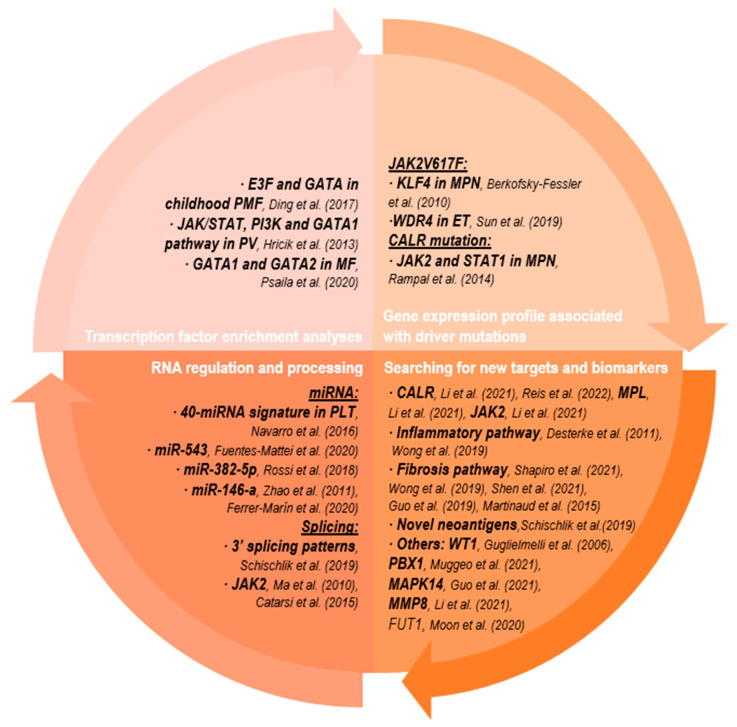
Main results of transcriptomic studies on MPNs. The corresponding references [49,56,57,58,59,60,61,62,63,64,65,66,67,68,69,70,71,72,73,74,75,76,77,78,79,80,81] are cited in the figure. Abbreviations: MF, myelofibrosis; PV, polycythemia vera; PMF, primary MF; MPN, myeloproliferative neoplasm; ET, essential thrombocythemia; PLT, platelets.

Although the elucidation of the molecular landscape of MPNs has been mainly focused on the detection of driver mutations, the presence of one of the three main driver mutations (*JAK2*, *CALR* and *MPL*) can lead to different phenotypes, demonstrating the influence of other factors in the development and progression of these neoplasms. Thus, the phenotype is not solely determined by the genomic profile, and disturbances in DNA transcription to mRNA or its translation to protein may be even more important. Here, we will describe some relevant studies that have endeavored to detect disturbances at the RNA level, whether at the gene expression level, splicing process or RNA regulation. The main studies explained in this section are summarized in Figure 1.

#### 2.2.1. Transcription Factor Enrichment Analyses

One of the studies that best represents the important role of transcriptomic biology was carried out by Ding and colleagues who observed and evaluated the different treatment response of a monozygotic twin pair with childhood PMF [56]. The brothers shared the same genetic information, lacked the presence of mutations in *JAK2, CALR* or *MPL* but harbored mutations in *SRSF2* and *SF3B1*, which could drive MF as well, as previously reported. Both patients received the same treatment, but unfortunately, only the younger brother responded. Notably, the mutational status did not change after treatment in either of them, suggesting that the differences in response should be explained by epigenomic or transcriptomic changes. In order to demonstrate this, the authors carried out a comparison of the transcriptomic profiles, which showed that the patient who responded presented with both the activation of the JAK/STAT signaling pathway and a clear influence of the cell cycle and apoptosis pathway through treatment. Particularly, after a transcription factor enrichment analysis, these authors showed that E3F and GATA were the transcription factors responsible for the differential expression of 40 genes after treatment, suggesting that their regulation could explain the differential treatment response between the brothers [56].

Indeed, other transcriptomic studies have also revealed the possible role of GATA1 in myeloproliferative neoplasms. One of them described the enrichment of two genes, *GPR56* and *RAB4a,* together with the lower expression of *TAL1* due to the increased phosphorylation of cytoplasmic kinases JAK/STAT, PI3K and GATA1 pathways in expanded erythroblasts from PV patients [57]. In the same line, a single cell RNA-seq study of 15 MF patients and 6 controls revealed differential expression patterns of the transcription factors, GATA1 and GATA2, among others, in the regulation of megakaryocyte–erythroid cell fate decisions [58].

#### 2.2.2. Gene Expression Profile Associated with Driver Mutation

Transcriptomic studies have also been carried out to evaluate the gene expression profile associated with driver mutations. For example, Berkofsy-Fessler and colleagues compared the transcriptional profile associated with the *JAK2* canonical mutation [59]. The authors identified a panel of 14 JAK2-dependent genes, such as *KLF4*, which is implied in the pluripotency maintaining of embryonic stem cells and a panel of 12 JAK2-independent genes. These two panels allowed them to distinguish between PV and controls and between ET or PMF and the control group. Although this work explains that aberrantly expressed genes in PV could not only be attributed to mutated *JAK2* signaling, many of them are deregulated by *JAK2-*independent unknown mechanisms, opening a new horizon in the pathogenesis of PV, since, as mentioned above, almost 95% of PV patients harbor *JAK2* mutations [5]. Other studies have related transcriptomic aberrations of mesenchymal stromal cells (MSCs) from patients with *JAK2*V617F-mutated ET patients to *WDR4* low expression with increased proliferation, reduced senescence and differentiation [60]. In contrast to both studies is a microarray gene expression analysis in 93 patients with MPNs (28 PV, 47 ET and 18 MF) in comparison to granulocytes of 11 age-matched normal donors; although this led to the identification of a characteristic gene expression profile related to JAK2 activation in MPN patients, it did not identify a specific expression signature derived from the *JAK2*-mutational state [61]. However, *CALR*-mutated patients presented a gene signature associated with activated JAK2 signaling (such as an upregulation in *JAK2* and *STAT1*) [61]. This fact supports the efficacy of JAK-targeted therapies in MPNs regardless of genotype. From another perspective, Zini and colleagues compared the transcriptomic profile of CD34+ cells from ET patients harboring *JAK2* or *CALR* mutations [82]. As expected, the authors described that different pathways were activated depending on the driver mutation (i.e., mTOR, MAPK/PI3K and MYC pathways), and despite of this, the same phenotype was shown, thus reinforcing the influence of transcriptomics on the disease.

#### 2.2.3. Searching for New Targets and Biomarkers

In addition to exploring gene expression programs derived from the presence of known mutations, another main goal of wide-ranging studies based on transcriptomic data is to discover new targets or biomarkers of progression in the disease [62]. As an example of this, several studies have shown the dysregulation of inflammatory pathways in MPN patients. An analysis of PMF CD34+ and megakaryocytes (MK) cell transcriptomes showed deregulation of the MAPK pathway along with *FLT3* expression [63]. A higher proportion of circulating FLT3+ CD34+ cells exhibited an increased MAPK effector phosphorylation independently of the *JAK2*V617F mutation in PMF patients. The activation of the FLT3 axis in PMF MK cell cultures induced the activation of the p38-MAPK cascade and overexpression of its targets (NFATC4, p53, AP-1 and IL-8), resulting in an inflammation context. As a consequence, reducing p38MAPK phosphorylation through FLT3 inhibition demonstrated the megakaryopoiesis process was improved [63].

Others have also identified a number of gene expression changes associated with inflammatory pathways and progression to fibrosis, distinguishing between pre-fibrotic MPNs and overtly fibrotic MPNs [64]. For example, the increased expression of several prefibrotic growth factors, matrix metalloproteinases, *VEGFA*, *IGFBP7* and cell cycle regulators (*CCND1*, *CCNA2*, *CCNB2* and *CCNF*) have defined a specific transcriptional signature in association with fibrosis development [65] and advanced stage disease [62]. In the same way, other authors also found the altered gene expression of cell cycle regulators *CCND1*, *H2AFX* and *CEP55* as a fibrosis-signature in MF patients [66]. Similarly, the transcriptional alterations of BM-MSC in PMF patients revealed high TGFβ1 signaling, driving to osteogenic potential [67]. Importantly, the inhibition of the TGFβ1 receptor abrogated the osteogenic differentiation of MSC, suggesting its use in combination with the inhibition of hematopoietic cell proliferation as a novel therapeutic strategy in MF. 

Apart from inflammatory or fibrotic pathways, other studies have found different novel targets. Using microarray analysis, the gene expression of CD34+ stem cells from the peripheral blood (PB) of idiopathic MF was compared to normal CD34+ controls [68]. The analyses revealed 174 differentially expressed genes, of which 8 (*CD9*, *GAS2*, *DLK1*, *CDH1*, *WT1*, *NFE2*, *HMGA2* and *CXCR4*) were differently expressed in the two studied populations. Among them, *WT1* expression was associated with a more active disease. Years later, Muggeo and colleagues interrogated the involvement of the pre-B-cell leukemia homeobox 1 (PBX1) in human MPNs [69]. Using a genetically modified knockout (KO) model, the authors found that when *PBX1* is removed, the expression levels of genes upregulated in MPNs were downregulated in the murine model. Therefore, PBX1 might act downstream of JAK2, proposing a new target subjected to novel therapies.

Finally, based on different approaches, the evaluation of the mutational profile of 113 MPN patients using transcriptomic data have helped to predict around 149 novel neoantigens in 62% of the evaluated patients, which could be very useful as a source for generating a personalized vaccine or developing an adaptive cell therapy [49].

Other studies identifying new candidate prognostic markers or potential therapeutic targets have described the possible role of MAPK14 in PV [70], EPB42, CALR, SLC4A1 and MPL [71], LCN2, JAK2, MMP8, CAMP, DEFA4, LTF, MPO, HBD, STAT4, EBF1 [72] in PMF, CDH6, EHD2, FUT1, KIF26A, LINC00346, PTPRN, SERF1A, SLC6A9 [73], and CALR in ET [74].

#### 2.2.4. RNA Regulation and Processing

The last part of this section includes transcriptomic studies that have evaluated alterations at the regulatory or processing level or RNA transcription. It is well known that microRNAs (miRNAs) regulate hematopoiesis, so their aberrant expression could prompt very diverse hematopoietic alterations. MiRNA deregulation in MPN patients has been known for years [83]. While some studies have described a 40-miRNA signature in platelets (PLT) of *JAK2*V617-negative [75], others have revealed the specific role of some miRNAs in MPNs. Thus, recent studies have revealed the role of miR-543 [76] and miR-382-5p [77] in MPNs. Furthermore, the deregulation of miR-146-a, a brake in NF-κB signaling, could drive a MF-like phenotype in a model of miR-146-a^KO^ mice [78]. In addition, data from our lab have shown that the rs2431697 TT genotype increases susceptibility to secondary MF and that it is a marker for early progression to secondary MF (independent of the *JAK2*V617F allele burden) [79].

Along the same lines, alterations in the 3′ untranslated regions (3′-UTRs), which are involved in mRNA stabilization and processing, may be implicated in the progression of many cancers such as MPNs [84]. Interestingly, new non-canonical *CALR* mutations in the 3′-UTRs, which are usually not detected by conventional techniques, have been described in *JAK2*V617/exon 12 mutation-negative MPN patients that resemble PV due to increased erythroid maturation [85]. By another way, as mutations in some splicing components are usual in myeloid malignancies and driver events in MPNs [6], some efforts have been focused on evaluating splicing aberrancies in splicing-mutated patients. In this sense, Schischlik and colleagues [49] demonstrated that MPN patients harboring *SF3B1* mutations provided distinct 3′ splicing patterns with 250 new genomic alterations. However, even in the absence of splicing mutations, splicing anomalies could occur. A notable example is the detection of the *JAK2* isoform lacking exon 14 in MPN patients [80,81].

### 2.3. Proteomic and Post-Translational Modifications

**Figure 2:**
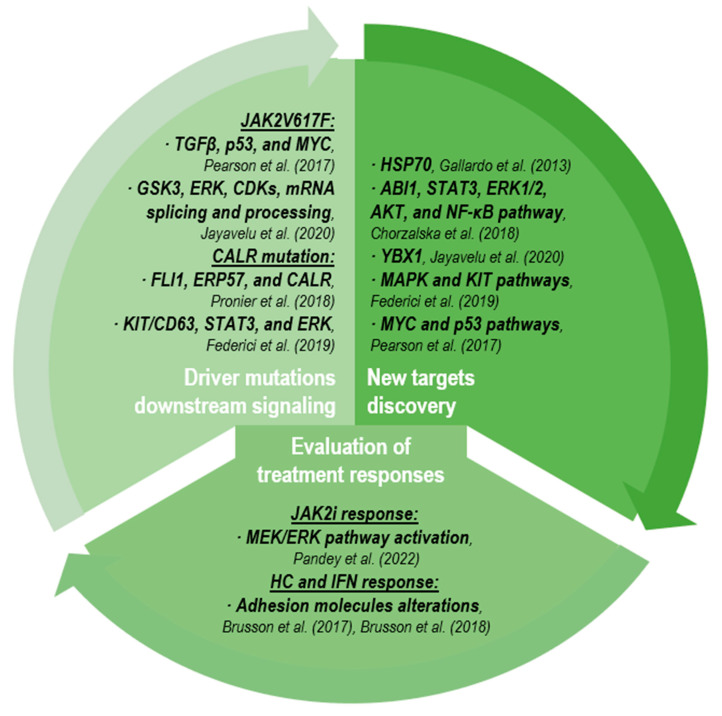
Main results of proteomic studies in MPNs. The corresponding references [86,87,88,89,90,91,92,93,94] are cited in the figure. Abbreviations: JAKi, JAK inhibitors; HC, hydroxycarbamide; IFN, interferon.

Similar to transcriptomic studies, proteomic analyses have mainly focused on studying molecular changes resulting from the presence of certain mutations, the discovery of new targets and the pharmacological responses. The main studies explained in this section are summarized in Figure 2.

#### 2.3.1. Driver Mutation Downstream Signaling

Since they were developed, proteomic profiling tools have helped to gain insights into the downstream molecular mechanisms activated by a particular mutation or pathway. The characterization of *JAK2*V617F signaling in MPNs is obviously of particular interest. The first study evaluating this was carried out by Mossuz and colleagues [95]. The authors investigated the influence of the *JAK2* canonical mutation in ET patients using mass spectrometry-based analysis and found that the mutation did not significantly impact the serum proteome, suggesting that its presence partially influenced the ET phenotype and that other additional factors may be involved. However, the cell proteome characterization from wild-type and V617F-mutated *JAK2* cells identified disruption changes in proteins from the TGFβ, p53 and MYC signaling as the most affected [86]. Later on, similar comparisons showed kinase motifs for glycogen synthase kinase-3 (GSK3), ERK and cyclin-dependent kinases (CDKs) enriched in *JAK2*-mutated cells, as well as increased phosphorylation of many proteins associated with the gene ontology terms, mRNA splicing and processing, as the main *JAK2*-mutated downstream signaling pathways [87]. 

In addition, protein interactome derived from *CALR* wild-type and mutated MPN cells were characterized, identifying higher CALR levels in the mutated cells and the increased recruitment of FLI1, ERP57 and CALR to the *MPL* promoter to enhance its transcription and, therefore, expression [88]. Similar approaches have helped to elucidate SCF (stem cell factor)/cKIT interplay in primary erythroid progenitors, using phosphoproteomic profiling of adult blood (AB), cord blood (CB) and polycythemia vera cells [89]. In this study, after SCF stimulation, the authors identified the activation of the noncanonical cKIT signaling pathway in PV patients, such as the constitutive association of cKIT and tetrasporin CD63, as well as higher levels of STAT3 and ERK and lower levels of AKT (versus AB). These observations reflected a greater involvement than expected of erythroid populations in PV.

#### 2.3.2. New Target Discovery

Proteomic analyses have also identified new targets as new therapeutic vulnerabilities in MPNs. In 2013, Gallardo and colleagues described the possible role of heat shock protein 70 (HSP70) as a key role in the proliferation and survival of the erythroid lineage in PV patients using two-dimensional gel electrophoresis (2D-DIGE) and mass spectrometry, suggesting its regulation as a potential therapeutic target [90]. In addition, new targets have been defined using murine models. In this sense, some researchers described the role of BM’s specific loss of *ABI1* as a driver of MF disease, which was validated in MPN patient samples [91]. In this MF murine model, the phosphoproteomic studies showed that STAT3, ERK1/2 and AKT phosphorylation was significantly increased via the hyperactivity of SFK (family Src kinase)/STAT3/NF-kB signaling pathways. Particularly, Abi-1^KO^ mice significantly presented with an overexpression of genes involved in the NF-κB pathway. On the other hand, Jayavelu and colleagues described, in their MPN murine model, the loss of *YBX1* as a sensitization mechanism for the inhibitory capacity of JAK inhibitors (JAKi), as genetic *YBX1* disruption helps diminish JAK2i-persistent cells [87]. Additionally, as YBX1 could be dependently phosphorylated via *JAK2*V617F and MEK/ERK pathways, a combination therapy based on ruxolitinib and trametinib (a MEK inhibitor) was evaluated in PDX (patient-derived xenograft) mice models, proving that cell growth was decreased and molecular remission reached compared to ruxolitinib-treated PDX mice. In the same direction, other authors have also suggested that MAPK or cKIT treatment in PV could enhance JAKi responses based on their results [89]. In addition, the simultaneous treatment of MYC and p53, based on JQ1 and nutlin combination, in *JAK2*V617F cells showed the potential of this dual therapy in MPNs [86]. 

#### 2.3.3. Evaluation of Treatment Responses

In addition, as previously noted [87], another advantage of proteomic studies is the evaluation of treatment responses. In this sense, a very recent study evaluated JAK inhibitor responses and described a central role for the activation of the MAPK survival pathways in persistent MPN cells, suggesting MEK/ERK pathway inhibition to get better treatment responses [92]. Particularly, Brusson and colleagues have previously reported that membranes of PV red blood cells presented an abnormal expression of several proteins, with some of them related to adhesion, such as Lu/BCAM (Lutheran/basal cell adhesion molecule) [93]. Later research from the same group [94] evaluated the effects of hydroxycarbamide (HC, hydroxyurea) and interferon-α (IFN) treatment in MPN patients applying proteomic studies. The authors described that both treatments diminished CALR levels, but neither of them up to the control levels. However, HC treatment enhanced the expression of proteins related to erythrocytes adhesion, such as Lu/BCAM and CD147, whereas IFN did not. Therefore, the results suggested that this altered expression maybe prompted a negative impact on vascular risk event occurrence, and HC treatment had adverse effects on red blood cell physiology. However, further studies assessing the actual risk of HC treatment in PV are needed.

## 3. Extrinsic Factors

Similarly to the intrinsic mechanisms involvement in the disease, extrinsic mechanisms may provide the signals that are necessary for the survival of MPN cells [10,96,97,98,99]. These mechanisms are also essential for MPN development, evolution or treatment resistance.

### 3.1. Microbiome

Since the first description of the relationship between Epstein–Barr virus and Burkitt’s lymphoma [100], other microorganisms infecting the human body have been linked to cancer development, such as *Helicobacter pylori* and human papilloma viruses [101]. 

Not only infections of pathogenic agents but also hosting species that live in symbiosis in the human body have shown growing evidence of their relationship with cancer, prompting scientific interest in recent decades. Indeed, there are studies estimating that in 2018, around 13% of global cancer incidence could be attributed to infection [102], as well as studies that relate microbial dysbiosis and cancer [103].

In recent years, microbiome dysbiosis has sparked interest in hematological diseases [104], and some studies evaluate the influence of microbiota on efficacy and treatment responses, including HSCT [105,106]. As certain bacterial species from the human microbiome secrete pathogenic products, some secreted molecules could provoke cell apoptosis, immune defense evasion, inflammatory processes or cancer evolution [107]. Therefore, microbiota composition could be responsible for patients’ inflammation state. As inflammation has been widely reviewed as a driver of MPN development [96], the dysbiosis of species related to this process could be particularly important in the disease context.

Specifically referring to MPNs, only a few studies have evaluated microbial dysbiosis. A first approach analyzed the microbial content in the PB and BM of 1870 newly diagnosed patients with myeloid malignancies (354 MPN patients) and compared it to 12 healthy controls [108]. Using deep DNA sequencing, the authors catalogued the bacterial, fungal and viral content in circulation, discovering dysbiosis in disease cases and different microbial fingerprints. The main differences were found in AML and MDS entities. Concretely in MPNs, the authors only suggested some kind of relationship between *JAK2* mutation presence and the circulating microbiome, but further studies are required. Either way, this study served as a baseline for future research analyzing microbiomes in myeloid malignancy patients.

As the connection between BM and the gut is well established [109], other researchers have also evaluated stool samples in their studies. Barone and colleagues [110] isolated the microbial DNA cargo of circulating MK- and PLT-derived extracellular vesicles due to their role in the inflammatory network [111], together with fecal samples in PV patients’ samples and carried out 16S rDNA V3-V4 region sequencing. They found a higher diversity and different composition of microbial content, with a potential role in inflammation, in extracellular vesicles from PV than in healthy controls. Although they could not identify a differential microbiome profile of the gut microbiota of PV and controls, they associated it with the cytotoxic therapy administered during the time of the study [110].

In contrast, the Canadian group lead by Fleischman [112,113] compared the gut microbiota of triplicate fecal samples from 25 MPN patients (PV, ET and secondary MF) and 25 healthy controls (co-inhabitants whenever possible). The researchers found 1.7% of microbial composition variance explained by the disease. In this sense, they detected lower reads of species, such as *Phascolarctobacterium*, associated with reduced inflammation, as well as higher levels of the *Prevotellaceae* family linked to chronic inflammatory conditions in MPNs. These results support the important role of inflammation as one of the main promoters of the disease. 

It is important to mention that most of the previous described studies [110,112,113] include patients under treatment that could modulate microbiome content, explaining some of the differences between studies. In addition, microbiome content is very individual and specific, and it changes according to diet and other external factors; therefore, establishing the correct controls is always challenging to obtain the best results. 

### 3.2. Other Extrinsic Factors

Other researchers have focused on the evaluation of the implication of environmental exposures in normal population driving to MPN. In this line, recent studies utilizing data from “The Danish Health Examination Survey” (DANHES) have associated smoking with MPN development. Among 75,896 included patients, 70 were newly diagnosed with MPNs (41 women and 29 men). The analysis identified smoking as a significant risk factor for the development of MPNs compared to patients in the general population in Denmark who had never smoked [114]. Indeed, it is known that smoking is a risk factor, especially for PV development [115], because it affects inflammation and oxidative stress [116].

In a similar approach, two different population-based cohorts, the Copenhagen General Population Study and the Copenhagen City Heart Study, were used to evaluate HDL levels and the cancer risk of several entities [117]. The authors identified that low HDL cholesterol and/or apolipoprotein A1 were associated with an increased risk of MPNs (HR = 1.66) and other malignancies, such as multiple myeloma, non-Hodgkin lymphoma, breast cancer, lung cancer and nervous system cancer [117].

## 4. Interaction between Intrinsic and Extrinsic Factors

Likely, one of the main mechanisms driving to MPNs is inflammation imbalance. Alterations in the process occur in both malignant and non-malignant clones as well. Therefore, not only intrinsic factors from MPN malignant cells but also tumor microenvironments might provoke a chronic inflammatory state that may eventually lead to the development of MPNs [96,118]. 

Nonetheless, other mechanisms not so widely known in MPNs, which are also important, are oxidative stress and cellular metabolism derived from malignant cells and tumor microenvironments [119]. Oxidative disturbances and metabolic alterations display a considerable position in tumorigenic processes [120,121], causing alterations in the normal cell fate decision processes. 

The oxidative status has been evaluated in PV, ET [122,123] and MF patients [124]. As with other myeloid malignancies, higher ROS levels and lower antioxidant capacity were observed in MPN patients compared to controls.

Metabolic dysregulation has also been well established in myeloid neoplasms, such as MDS [125] and AML [126], where the survival of leukemic stem cells (LSCs) has been explained by the alteration of different sources for energy production [127,128]. In MPNs, mutant *JAK2* has been described to enhance the expression of glycolytic enzymes that sustain cell growth [129]. In vivo experiments have shown that *JAK2*-mutant hematopoietic cells displayed metabolic alterations essential for the pathogenesis of these neoplasms, leading to hypoglycemia, adipose tissue atrophy and early mortality [130] that could be reversed in the murine model using 3-(3-pyridinyl)-1-(4pyridinyl)-2-propen-1-one, which inhibits Pfkfb3, a key regulator of glycolysis. A similar approach in mice carrying both *NRAS* and *EZH2* mutations showed that the hyperactivation of branched-chain amino acid (BCAA) metabolism due to the aberrant activation of BCAT1 was responsible for more aggressive MPNs with rapid progression to the acute leukemia phase, suggesting dietary BCAA restriction or the use of BCAT1 inhibitors in *EZH2*-mutated myeloid neoplasms [131]. These results reinforce the potential for targeting metabolism in mutant MPN cells [132].

On the other hand, the interaction between malignant and non-malignant clones in MPN patients showed different metabolic profiles in PV and ET patients compared to healthy controls, reflecting the energetic demands for fast proliferation in MPN patients [133]. Nevertheless, further studies on metabolism dysregulation in MPNs are needed to determine whether specific targeted drugs may be useful as a novel treatment for the disease [10].

**Figure 3:**
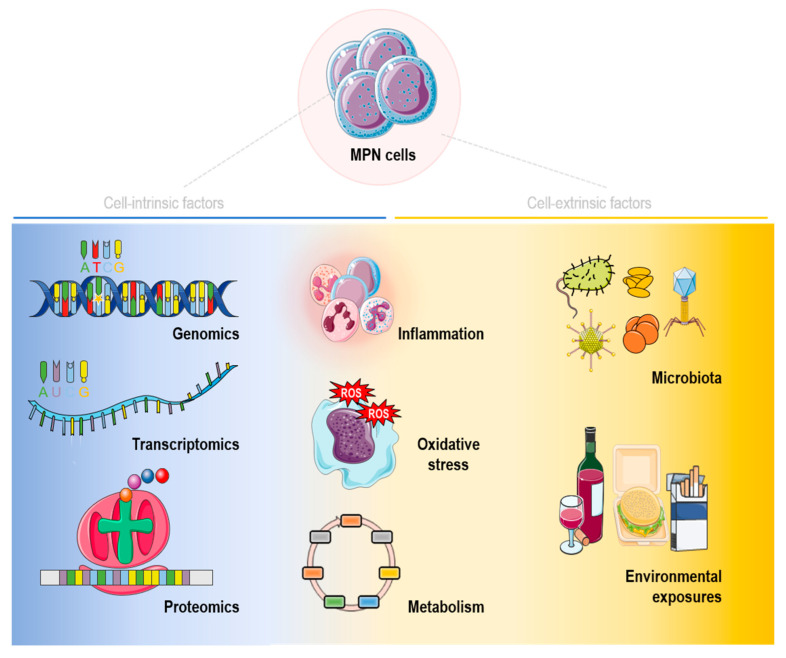
General overview of cell-intrinsic and -extrinsic factors and their interaction, all affecting MPNs.

A general diagram of the interaction between intrinsic and extrinsic factors that may be relevant for MPNs is shown in Figure 3.

## 5. Conclusions

In summary, although MPNs are classically associated with the alteration of the JAK/STAT pathway, it is not the only signaling pathway altered in patients. A clear example of this is the lack of response to JAK inhibitors. In fact, despite the considerable progress which has been made in the molecular understanding of the disease thanks to high-throughput studies, they have raised many questions to be addressed in the next years. 

As described throughout this review, MPNs are not only influenced by intrinsic factors that can be localized in the clonal malignant cells but also, very importantly, by other cell-extrinsic factors, with many of them yet described as hallmarks of cancer. Ultimately, the interaction between all these factors likely contributes to the origin, evolution and progression of these neoplasms. The contribution and weight of each of these factors, as well as their translatability to the clinical setting, will be seen in the coming years in the context of increasingly personalized medicine.

## References

[1] Khoury J.D., Solary E., Abla O., Akkari Y., Alaggio R., Apperley J.F., Bejar R., Berti E., Busque L., Chan J.K.C. (2022). The 5th edition of the World Health Organization Classification of Haematolymphoid Tumours: Myeloid and Histiocytic/Dendritic Neoplasms. Leukemia.

[2] Arber D.A., Orazi A., Hasserjian R.P., Borowitz M.J., Calvo K.R., Kvasnicka H.-M., Wang S.A., Bagg A., Barbui T., Branford S. (2022). International Consensus Classification of Myeloid Neoplasms and Acute Leukemias: Integrating morphologic, clinical, and genomic data. Blood.

[3] Rolles B., Mullally A. (2022). Molecular Pathogenesis of Myeloproliferative Neoplasms. Curr. Hematol. Malign Rep..

[4] Rumi E., Cazzola M. (2017). Diagnosis, risk stratification, and response evaluation in classical myeloproliferative neoplasms. Blood.

[5] Vainchenker W., Kralovics R. (2017). Genetic basis and molecular pathophysiology of classical myeloproliferative neoplasms. Blood.

[6] Grinfeld J., Nangalia J., Baxter E.J., Wedge D.C., Angelopoulos N., Cantrill R., Godfrey A.L., Papaemmanuil E., Gundem G., MacLean C. (2018). Classification and Personalized Prognosis in Myeloproliferative Neoplasms. N. Engl. J. Med..

[7] Hultcrantz M., Kristinsson S.Y., Andersson T.M.-L., Landgren O., Eloranta S., Derolf R., Dickman P.W., Björkholm M. (2012). Patterns of Survival Among Patients With Myeloproliferative Neoplasms Diagnosed in Sweden From 1973 to 2008: A Population-Based Study. J. Clin. Oncol..

[8] Hultcrantz M., Wilkes S.R., Kristinsson S.Y., Andersson T.M.-L., Derolf R., Eloranta S., Samuelsson J., Landgren O., Dickman P.W., Lambert P.C. (2015). Risk and Cause of Death in Patients Diagnosed With Myeloproliferative Neoplasms in Sweden Between 1973 and 2005: A Population-Based Study. J. Clin. Oncol..

[9] McLornan D.P., Yakoub-Agha I., Robin M., Chalandon Y., Harrison C.N., Kroger N. (2019). State-of-the-art review: Allogeneic stem cell transplantation for myelofibrosis in 2019. Haematologica.

[10] Sharma V., Wright K.L., Epling-Burnette P.K., Reuther G.W. (2020). Metabolic Vulnerabilities and Epigenetic Dysregulation in Myeloproliferative Neoplasms. Front. Immunol..

[11] Zoi K., Cross N. (2017). Genomics of Myeloproliferative Neoplasms. J. Clin. Oncol..

[12] Baxter E.J., Scott L.M., Campbell P.J., East C., Fourouclas N., Swanton S., Vassiliou G.S., Bench A.J., Boyd E.M., Curtin N. (2005). Acquired mutation of the tyrosine kinase JAK2 in human myeloproliferative disorders. Lancet.

[13] James C., Ugo V., Le Couédic J.-P., Staerk J., Delhommeau F., Lacout C., Garçon L., Raslova H., Berger R., Bennaceur-Griscelli A. (2005). A unique clonal JAK2 mutation leading to constitutive signalling causes polycythaemia vera. Nature.

[14] Levine R.L., Wadleigh M., Cools J., Ebert B.L., Wernig G., Huntly B.J., Boggon T.J., Wlodarska I., Clark J.J., Moore S. (2005). Activating mutation in the tyrosine kinase JAK2 in polycythemia vera, essential thrombocythemia, and myeloid metaplasia with myelofibrosis. Cancer Cell.

[15] Kralovics R., Passamonti F., Buser A.S., Teo S.-S., Tiedt R., Passweg J.R., Tichelli A., Cazzola M., Skoda R.C. (2005). A Gain-of-Function Mutation of *JAK2* in Myeloproliferative Disorders. N. Engl. J. Med..

[16] Pikman Y., Lee B.H., Mercher T., McDowell E., Ebert B.L., Gozo M., Cuker A., Wernig G., Moore S., Galinsky I. (2006). MPLW515L Is a Novel Somatic Activating Mutation in Myelofibrosis with Myeloid Metaplasia. PLOS Med..

[17] Klampfl T., Gisslinger H., Harutyunyan A.S., Nivarthi H., Rumi E., Milosevic J.D., Them N.C.C., Berg T., Gisslinger B., Pietra D. (2013). Somatic Mutations of Calreticulin in Myeloproliferative Neoplasms. N. Engl. J. Med..

[18] Nangalia J., Massie C.E., Baxter E.J., Nice F.L., Gundem G., Wedge D.C., Avezov E., Li J., Kollmann K., Kent D.G. (2013). SomaticCALRMutations in Myeloproliferative Neoplasms with Nonmutated JAK2. N. Engl. J. Med..

[19] Jacquelin S., Kramer F., Mullally A., Lane S.W. (2020). Murine Models of Myelofibrosis. Cancers.

[20] Scott L.M., Tong W., Levine R.L., Scott M.A., Beer P.A., Stratton M.R., Futreal P.A., Erber W.N., McMullin M.F., Harrison C.N. (2007). *JAK2*Exon 12 Mutations in Polycythemia Vera and Idiopathic Erythrocytosis. N. Engl. J. Med..

[21] Guglielmelli P., Loscocco G.G., Mannarelli C., Rossi E., Mannelli F., Ramundo F., Coltro G., Betti S., Maccari C., Ceglie S. (2021). JAK2V617F variant allele frequency >50% identifies patients with polycythemia vera at high risk for venous thrombosis. Blood Cancer J..

[22] Loscocco G.G., Guglielmelli P., Gangat N., Rossi E., Mannarelli C., Betti S., Maccari C., Ramundo F., Jadoon Y., Gesullo F. (2021). Clinical and molecular predictors of fibrotic progression in essential thrombocythemia: A multicenter study involving 1607 patients. Am. J. Hematol..

[23] Defour J.-P., Chachoua I., Pecquet C., Constantinescu S. (2015). Oncogenic activation of MPL/thrombopoietin receptor by 17 mutations at W515: Implications for myeloproliferative neoplasms. Leukemia.

[24] Boyd E.M., Bench A.J., Goday-Fernández A., Anand S., Vaghela K.J., Beer P., Scott M.A., Bareford D., Green A.R., Huntly B. (2010). Clinical utility of routine *MPL* exon 10 analysis in the diagnosis of essential thrombocythaemia and primary myelofibrosis. Br. J. Haematol..

[25] Ding J., Komatsu H., Wakita A., Kato-Uranishi M., Ito M., Satoh A., Tsuboi K., Nitta M., Miyazaki H., Iida S. (2004). Familial essential thrombocythemia associated with a dominant-positive activating mutation of the c-MPL gene, which encodes for the receptor for thrombopoietin. Blood.

[26] Jia R., Kralovics R. (2019). Progress in elucidation of molecular pathophysiology of myeloproliferative neoplasms and its application to therapeutic decisions. Int. J. Hematol..

[27] Mansier O., Paz D.L., Ianotto J.-C., Le Bris Y., Chauveau A., Boyer F., Conejero C., Fitoussi O., Riou J., Adiko D. (2018). Clinical and biological characterization of MPN patients harboring two driver mutations, a French intergroup of myeloproliferative neoplasms (FIM) study. Am. J. Hematol..

[28] Hermange G., Rakotonirainy A., Bentriou M., Tisserand A., El-Khoury M., Girodon F., Marzac C., Vainchenker W., Plo I., Cournède P.-H. (2022). Inferring the initiation and development of myeloproliferative neoplasms. Proc. Natl. Acad. Sci. USA.

[29] Sousos N., Leathlobhair M.N., Karali C.S., Louka E., Bienz N., Royston D., Clark S.-A., Hamblin A., Howard K., Mathews V. (2022). In utero origin of myelofibrosis presenting in adult monozygotic twins. Nat. Med..

[30] Van Egeren D., Escabi J., Nguyen M., Liu S., Reilly C.R., Patel S., Kamaz B., Kalyva M., DeAngelo D.J., Galinsky I. (2021). Reconstructing the Lineage Histories and Differentiation Trajectories of Individual Cancer Cells in Myeloproliferative Neoplasms. Cell Stem Cell.

[31] Williams N., Lee J., Moore L., Baxter E.J., Hewinson J., Dawson K.J., Menzies A., Godfrey A.L., Green A.R., Campbell P.J. (2020). Phylogenetic Reconstruction of Myeloproliferative Neoplasm Reveals Very Early Origins and Lifelong Evolution. Biol. Med. BioRxiv.

[32] Langabeer S.E. (2016). Chasing down the triple-negative myeloproliferative neoplasms: Implications for molecular diagnostics. Jak-Stat.

[33] Alimam S., Villiers W., Dillon R., Simpson M., Runglall M., Smith A., Chatzikyriakou P., Lavender P., Kanda A., Mills K. (2021). Patients with triple-negative, *JAK2*V617F- and *CALR*-mutated essential thrombocythemia share a unique gene expression signature. Blood Adv..

[34] Lundberg P., Karow A., Nienhold R., Looser R., Hao-Shen H., Nissen I., Girsberger S., Lehmann T., Passweg J., Stern M. (2014). Clonal evolution and clinical correlates of somatic mutations in myeloproliferative neoplasms. Blood.

[35] Lee J., Godfrey A.L., Nangalia J. (2020). Genomic heterogeneity in myeloproliferative neoplasms and applications to clinical practice. Blood Rev..

[36] Jaiswal S., Fontanillas P., Flannick J., Manning A., Grauman P.V., Mar B.G., Lindsley R.C., Mermel C.H., Burtt N., Chavez A. (2014). Age-Related Clonal Hematopoiesis Associated with Adverse Outcomes. N. Engl. J. Med..

[37] Jaiswal S., Natarajan P., Silver A.J., Gibson C.J., Bick A.G., Shvartz E., McConkey M., Gupta N., Gabriel S., Ardissino D. (2017). Clonal Hematopoiesis and Risk of Atherosclerotic Cardiovascular Disease. N. Engl. J. Med..

[38] Segura-Díaz A., Stuckey R., Florido Y., González-Martín J.M., López-Rodríguez J.F., Sánchez-Sosa S., González-Pérez E., Perdomo M.N.S.S., Perera M.D.M., De la Iglesia S. (2020). Thrombotic Risk Detection in Patients with Polycythemia Vera: The Predictive Role of DNMT3A/TET2/ASXL1 Mutations. Cancers.

[39] Guglielmelli P., Gangat N., Coltro G., Lasho T.L., Loscocco G.G., Finke C.M., Morsia E., Sordi B., Szuber N., Hanson C.A. (2021). Mutations and thrombosis in essential thrombocythemia. Blood Cancer J..

[40] Bartels S., Vogtmann J., Schipper E., Büsche G., Schlue J., Lehmann U., Kreipe H. (2021). Combination of myeloproliferative neoplasm driver gene activation with mutations of splice factor or epigenetic modifier genes increases risk of rapid blastic progression. Eur. J. Haematol..

[41] Jongen-Lavrencic M., Grob T., Hanekamp D., Kavelaars F.G., al Hinai A., Zeilemaker A., Erpelinck-Verschueren C.A., Gradowska P.L., Meijer R., Cloos J. (2018). Molecular Minimal Residual Disease in Acute Myeloid Leukemia. N. Engl. J. Med..

[42] Morishita S., Hashimoto Y., Furuya C., Edahiro Y., Ochiai T., Shirane S., Inano T., Yasuda H., Ando M., Araki M. (2022). Non-driver gene mutation analysis in a large cohort of polycythemia vera and essential thrombocythemia. Eur. J. Haematol..

[43] Tefferi A., Guglielmelli P., Lasho T.L., Rotunno G., Finke C., Mannarelli C., Belachew A., Pancrazzi A., Wassie E., Ketterling R. (2014). CALR and ASXL1 mutations-based molecular prognostication in primary myelofibrosis: An international study of 570 patients. Leukemia.

[44] Guglielmelli P., Biamonte F., Score J., Hidalgo-Curtis C., Cervantes F., Maffioli M., Fanelli T., Ernst T., Winkelman N., Jones A.V. (2011). EZH2 mutational status predicts poor survival in myelofibrosis. Blood.

[45] Vannucchi A.M., Lasho T.L., Guglielmelli P., Biamonte F., Pardanani A., Pereira A., Finke C., Score J., Gangat N., Mannarelli C. (2013). Mutations and prognosis in primary myelofibrosis. Leukemia.

[46] Guglielmelli P., Lasho T.L., Rotunno G., Mudireddy M., Mannarelli C., Nicolosi M., Pacilli A., Pardanani A., Rumi E., Rosti V. (2018). MIPSS70: Mutation-Enhanced International Prognostic Score System for Transplantation-Age Patients With Primary Myelofibrosis. J. Clin. Oncol..

[47] Shiozawa Y., Malcovati L., Gallì A., Sato-Otsubo A., Kataoka K., Sato Y., Watatani Y., Suzuki H., Yoshizato T., Yoshida K. (2018). Aberrant splicing and defective mRNA production induced by somatic spliceosome mutations in myelodysplasia. Nat. Commun..

[48] Hautin M., Mornet C., Chauveau A., Bernard D.G., Corcos L., Lippert E. (2020). Splicing Anomalies in Myeloproliferative Neoplasms: Paving the Way for New Therapeutic Venues. Cancers.

[49] Schischlik F., Jäger R., Rosebrock F., Hug E., Schuster M., Holly R., Fuchs E., Milosevic Feenstra J.D., Bogner E., Gisslinger B. (2019). Mutational landscape of the transcriptome offers putative targets for immunotherapy of myeloproliferative neoplasms. Blood.

[50] Tefferi A., Guglielmelli P., Lasho T.L., Coltro G., Finke C.M., Loscocco G.G., Sordi B., Szuber N., Rotunno G., Pacilli A. (2020). Mutation-enhanced international prognostic systems for essential thrombocythaemia and polycythaemia vera. Br. J. Haematol..

[51] Loscocco G.G., Guglielmelli P., Mannelli F., Mora B., Mannarelli C., Rotunno G., Pancani F., Maccari C., Bartalucci N., Romagnoli S. (2022). *SF3B1* mutations in primary and secondary myelofibrosis: Clinical, molecular and prognostic correlates. Am. J. Hematol..

[52] Lasho T.L., Finke C.M., Hanson C.A., Jimma T., Knudson R.A., Ketterling R.P., Pardanani A., Tefferi A. (2012). SF3B1 mutations in primary myelofibrosis: Clinical, histopathology and genetic correlates among 155 patients. Leukemia.

[53] Zhang S.-J., Rampal R., Manshouri T., Patel J., Mensah N., Kayserian A., Hricik T., Heguy A., Hedvat C., Gönen M. (2012). Genetic analysis of patients with leukemic transformation of myeloproliferative neoplasms shows recurrent SRSF2 mutations that are associated with adverse outcome. Blood.

[54] Bernard E., Nannya Y., Hasserjian R.P., Devlin S.M., Tuechler H., Medina-Martinez J.S., Yoshizato T., Shiozawa Y., Saiki R., Malcovati L. (2020). Implications of TP53 allelic state for genome stability, clinical presentation and outcomes in myelodysplastic syndromes. Nat. Med..

[55] Rodriguez-Meira A., Rahman H., Norfo R., Wen W., Chédeville A., O’Sullivan J., Wang G., Paterson A., Louka E., Brierley C.K. (2021). Single-Cell Multi-Omics Reveals the Genetic, Cellular and Molecular Landscape of *TP53* Mutated Leukemic Transformation in MPN. Blood.

[56] Ding N., Zhang Z., Yang W., Ren L., Zhang Y., Zhang J., Li Z., Zhang P., Zhu X., Chen X. (2017). Transcriptome Analysis of Monozygotic Twin Brothers with Childhood Primary Myelofibrosis. Genom. Proteom. Bioinform..

[57] Hricik T., Federici G., Zeuner A., Alimena G., Tafuri A., Tirelli V., Varricchio L., Masiello F., Ciaffoni F., Vaglio S. (2013). Transcriptomic and phospho-proteomic analyzes of erythroblasts expanded in vitro from normal donors and from patients with polycythemia vera. Am. J. Hematol..

[58] Psaila B., Wang G., Rodriguez-Meira A., Li R., Heuston E.F., Murphy L., Yee D., Hitchcock I.S., Sousos N., O’Sullivan J. (2020). Single-Cell Analyses Reveal Megakaryocyte-Biased Hematopoiesis in Myelofibrosis and Identify Mutant Clone-Specific Targets. Mol. Cell.

[59] Berkofsky-Fessler W., Buzzai M., Kim M.K.-H., Fruchtman S., Najfeld V., Min D.-J., Costa F.F., Bischof J.M., Soares M.B., McConnell M.J. (2010). Transcriptional Profiling of Polycythemia Vera Identifies Gene Expression Patterns Both Dependent and Independent from the Action of JAK2V617F. Clin. Cancer Res..

[60] Sun T., Ju M., Dai X., Dong H., Gu W., Gao Y., Fu R., Liu X., Huang Y., Liu W. (2019). Multilevel defects in the hematopoietic niche in essential thrombocythemia. Haematologica.

[61] Rampal R., Al-Shahrour F., Abdel-Wahab O., Patel J.P., Brunel J.-P., Mermel C.H., Bass A.J., Pretz J., Ahn J., Hricik T. (2014). Integrated genomic analysis illustrates the central role of JAK-STAT pathway activation in myeloproliferative neoplasm pathogenesis. Blood.

[62] Shapiro S., Murphy L., Psaila B. (2021). Message in a platelet: Decoding platelet transcriptomes in myeloproliferative neoplasms. Cell Rep. Med..

[63] Desterke C., Bilhou-Nabéra C., Guerton B., Martinaud C., Tonetti C., Clay D., Guglielmelli P., Vannucchi A., Bordessoule D., Hasselbalch H. (2011). FLT3-Mediated p38–MAPK Activation Participates in the Control of Megakaryopoiesis in Primary Myelofibrosis. Cancer Res.

[64] Wong W.J., Baltay M., Getz A., Fuhrman K., Aster J.C., Hasserjian R.P., Pozdnyakova O. (2019). Gene expression profiling distinguishes prefibrotic from overtly fibrotic myeloproliferative neoplasms and identifies disease subsets with distinct inflammatory signatures. PLoS ONE.

[65] Shen Z., Du W., Perkins C., Fechter L., Natu V., Maecker H., Rowley J., Gotlib J., Zehnder J., Krishnan A. (2021). Platelet transcriptome identifies progressive markers and potential therapeutic targets in chronic myeloproliferative neoplasms. Cell Rep. Med..

[66] Guo B.B., Linden M.D., Fuller K.A., Phillips M., Mirzai B., Wilson L., Chuah H., Liang J., Howman R., Grove C.S. (2019). Platelets in myeloproliferative neoplasms have a distinct transcript signature in the presence of marrow fibrosis. Br. J. Haematol..

[67] Martinaud C., Desterke C., Konopacki J., Pieri L., Torossian F., Golub R., Schmutz S., Anginot A., Guerton B., Rochet N. (2015). Osteogenic Potential of Mesenchymal Stromal Cells Contributes to Primary Myelofibrosis. Cancer Res..

[68] Guglielmelli P., Zini R., Bogani C., Salati S., Pancrazzi A., Bianchi E., Mannelli F., Ferrari S., Le Bousse-Kerdilès M.-C., Bosi A. (2006). Molecular Profiling of CD34+ Cells in Idiopathic Myelofibrosis Identifies a Set of Disease-Associated Genes and Reveals the Clinical Significance of Wilms’ Tumor Gene 1 (*WT1*). Stem Cells.

[69] Muggeo S., Crisafulli L., Uva P., Fontana E., Ubezio M., Morenghi E., Colombo F.S., Rigoni R., Peano C., Vezzoni P. (2021). PBX1-directed stem cell transcriptional program drives tumor progression in myeloproliferative neoplasm. Stem Cell Rep..

[70] Guo C., Gao Y.-Y., Ju Q.-Q., Wang M., Zhang C.-X., Gong M., Li Z.-L. (2021). MAPK14 over-expression is a transcriptomic feature of polycythemia vera and correlates with adverse clinical outcomes. J. Transl. Med..

[71] Li W., Zhao Y., Wang D., Ding Z., Li C., Wang B., Xue X., Ma J., Deng Y., Liu Q. (2021). Transcriptome research identifies four hub genes related to primary myelofibrosis: A holistic research by weighted gene co-expression network analysis. Aging.

[72] Li W., Yuan B., Zhao Y., Lu T., Zhang S., Ding Z., Wang D., Zhong S., Gao G., Yan M. (2021). Transcriptome profiling reveals target in primary myelofibrosis together with structural biology study on novel natural inhibitors regarding JAK2. Aging.

[73] Moon K.C., Gim J.-A., Kim D.S., Choi C.W., Yoon J., Yoon S.-Y. (2020). Total Platelet Transcriptomics and Its Network Analysis by RNA-Seq and miRNA-Seq and PCA Application in Essential Thrombocythaemia. Acta Haematol..

[74] Reis E., Buonpane R., Celik H., Marty C., Lei A., Jobe F., Rupar M., Zhang Y., DiMatteo D., Awdew R. (2022). Discovery of INCA033989, a Monoclonal Antibody That Selectively Antagonizes Mutant Calreticulin Oncogenic Function in Myeloproliferative Neoplasms (MPNs). Blood.

[75] Navarro A., Pairet S., Álvarez-Larrán A., Pons A., Ferrer G., Longarón R., Fernández-Rodríguez C., Camacho L., Monzó M., Besses C. (2016). miR-203 and miR-221 regulate SOCS1 and SOCS3 in essential thrombocythemia. Blood Cancer J..

[76] Fuentes-Mattei E., Bayraktar R., Manshouri T., Silva A.M., Ivan C., Gulei D., Fabris L., Amaral N.S.D., Mur P., Perez C. (2020). miR-543 regulates the epigenetic landscape of myelofibrosis by targeting TET1 and TET2. J. Clin. Investig..

[77] Rossi C., Zini R., Rontauroli S., Ruberti S., Prudente Z., Barbieri G., Bianchi E., Salati S., Genovese E., Bartalucci N. (2018). Role of TGF -β1/miR-382-5p/ SOD 2 axis in the induction of oxidative stress in CD 34+ cells from primary myelofibrosis. Mol. Oncol..

[78] Zhao J.L., Rao D.S., Boldin M.P., Taganov K.D., O’Connell R.M., Baltimore D. (2011). NF-κB dysregulation in microRNA-146a–deficient mice drives the development of myeloid malignancies. Proc. Natl. Acad. Sci. USA.

[79] Ferrer-Marín F., Arroyo A.B., Bellosillo B., Cuenca E.J., Zamora L., Hernández-Rivas J.M., Hernández-Boluda J.C., Fernandez-Rodriguez C., Luño E., Hernandez C.G. (2020). miR-146a rs2431697 identifies myeloproliferative neoplasm patients with higher secondary myelofibrosis progression risk. Leukemia.

[80] Ma W., Kantarjian H., Zhang X., Wang X., Zhang Z., Yeh C.-H., O’Brien S., Giles F., Bruey J.M., Albitar M. (2010). JAK2 Exon 14 Deletion in Patients with Chronic Myeloproliferative Neoplasms. PLoS ONE.

[81] Catarsi P., Rosti V., Morreale G., Poletto V., Villani L., Bertorelli R., Pedrazzini M., Zorzetto M., Barosi G. (2015). AGIMM Investigators JAK2 Exon 14 Skipping in Patients with Primary Myelofibrosis: A Minor Splice Variant Modulated by the JAK2-V617F Allele Burden. PLoS ONE.

[82] Zini R., Guglielmelli P., Pietra D., Rumi E., Rossi C., Rontauroli S., Genovese E., Fanelli T., Calabresi L., Bianchi E. (2017). CALR mutational status identifies different disease subtypes of essential thrombocythemia showing distinct expression profiles. Blood Cancer J..

[83] Zhan H., Cardozo C., Raza A. (2013). MicroRNAs in myeloproliferative neoplasms. Br. J. Haematol..

[84] Wei W., Gao W., Li Q., Liu Y., Chen H., Cui Y., Sun Z., Liu Z. (2022). Comprehensive characterization of posttranscriptional impairment-related 3′-UTR mutations in 2413 whole genomes of cancer patients. NPJ Genom. Med..

[85] Quattrocchi A., Quattrocchi A., Maiorca C., Maiorca C., Billi M., Billi M., Tomassini S., Tomassini S., De Marinis E., De Marinis E. (2020). Genetic lesions disrupting calreticulin 3′-untranslated region in JAK2 mutation-negative polycythemia vera. Am. J. Hematol..

[86] Pearson S., Williamson A.J.K., Blance R., Somervaille T.C.P., Taylor S., Azadbakht N., Whetton A.D., Pierce A. (2017). Proteomic analysis of JAK2V617F-induced changes identifies potential new combinatorial therapeutic approaches. Leukemia.

[87] Jayavelu A.K., Schnöder T.M., Perner F., Herzog C., Meiler A., Krishnamoorthy G., Huber N., Mohr J., Edelmann-Stephan B., Austin R. (2020). Splicing factor YBX1 mediates persistence of JAK2-mutated neoplasms. Nature.

[88] Pronier E., Cifani P., Merlinsky T.R., Berman K.B., Somasundara A.V.H., Rampal R.K., Lacava J., Wei K.E., Pastore F., Maag J.L. (2018). Targeting the CALR interactome in myeloproliferative neoplasms. J. Clin. Investig..

[89] Federici G., Varricchio L., Martelli F., Falchi M., Picconi O., Francescangeli F., Contavalli P., Girelli G., Tafuri A., Petricoin E.F.I. (2019). Phosphoproteomic Landscaping Identifies Non-canonical cKIT Signaling in Polycythemia Vera Erythroid Progenitors. Front. Oncol..

[90] Gallardo M., Barrio S., Fernandez M., Paradela A., Arenas A., Toldos O., Ayala R., Albizua E., Jimenez A., Redondo S. (2013). Proteomic analysis reveals heat shock protein 70 has a key role in polycythemia Vera. Mol. Cancer.

[91] Chorzalska A., Morgan J., Ahsan N., Treaba D.O., Olszewski A.J., Petersen M., Kingston N., Cheng Y., Lombardo K., Schorl C. (2018). Bone marrow–specific loss of ABI1 induces myeloproliferative neoplasm with features resembling human myelofibrosis. Blood.

[92] Pandey G., Kuykendall A.T., Reuther G.W. (2022). JAK2 inhibitor persistence in MPN: Uncovering a central role of ERK activation. Blood Cancer J..

[93] Brusson M., Cochet S., Leduc M., Guillonneau F., Mayeux P., Peyrard T., Chomienne C., Le Van Kim C., Cassinat B., Kiladjian J.-J. (2017). Enhanced calreticulin expression in red cells of polycythemia vera patients harboring the *JAK2*^V617F^ mutation. Haematologica.

[94] Brusson M., De Grandis M., Cochet S., Bigot S., Marin M., LeDuc M., Guillonneau F., Mayeux P., Peyrard T., Chomienne C. (2018). Impact of hydroxycarbamide and interferon-α on red cell adhesion and membrane protein expression in polycythemia vera. Haematologica.

[95] Mossuz P., Arlotto M., Hermouet S., Bouamrani A., Lippert E., Girodon F., Dobo I., Vincent P., Cahn J.Y., Berger F. (2008). Proteomic study of the impact of the JAK2–V617F mutation on the phenotype of essential thrombocythemia. Exp. Hematol..

[96] Koschmieder S., Chatain N. (2020). Role of inflammation in the biology of myeloproliferative neoplasms. Blood Rev..

[97] Ramanathan G., Fleischman A.G. (2020). The Microenvironment in Myeloproliferative Neoplasms. Hematol. Clin. North Am..

[98] Catani L., Cavo M., Palandri F. (2021). The Power of Extracellular Vesicles in Myeloproliferative Neoplasms: “Crafting” a Microenvironment That Matters. Cells.

[99] Zhan H., Kaushansky K. (2020). The Hematopoietic Microenvironment in Myeloproliferative Neoplasms: The Interplay Between Nature (Stem Cells) and Nurture (the Niche). Tumor Microenviron. Hematop. Cells Part B.

[100] Epstein M., Achong B., Barr Y. (1964). Virus Particles in Cultured Lymphoblasts from Burkitt’s Lymphoma. Lancet.

[101] Azevedo M.M., Pina-Vaz C., Baltazar F. (2020). Microbes and Cancer: Friends or Faux?. Int. J. Mol. Sci..

[102] de Martel C., Georges D., Bray F., Ferlay J., Clifford G.M. (2020). Global burden of cancer attributable to infections in 2018: A worldwide incidence analysis. Lancet Glob. Health.

[103] Sobhani I., Tap J., Roudot-Thoraval F., Roperch J.P., Letulle S., Langella P., Corthier G., Van Nhieu J.T., Furet J.P. (2011). Microbial Dysbiosis in Colorectal Cancer (CRC) Patients. PLoS ONE.

[104] Uribe-Herranz M., Klein-González N., Rodríguez-Lobato L.G., Juan M., de Larrea C.F. (2021). Gut Microbiota Influence in Hematological Malignancies: From Genesis to Cure. Int. J. Mol. Sci..

[105] Elinav E., Garrett W.S., Trinchieri G., Wargo J. (2019). The cancer microbiome. Nat. Rev. Cancer.

[106] Wolfe A.E., Markey K.A. (2022). The contribution of the intestinal microbiome to immune recovery after HCT. Front. Immunol..

[107] Goodman B., Gardner H. (2018). The microbiome and cancer. J. Pathol..

[108] Woerner J., Huang Y., Hutter S., Gurnari C., Sánchez J.M.H., Wang J., Huang Y., Schnabel D., Aaby M., Xu W. (2022). Circulating microbial content in myeloid malignancy patients is associated with disease subtypes and patient outcomes. Nat. Commun..

[109] Santisteban M., Kim S., Pepine C., Raizada M.K. (2016). Brain–Gut–Bone Marrow Axis. Circ. Res..

[110] Barone M., Barone M., Ricci F., Auteri G., Corradi G., Fabbri F., Papa V., Bandini E., Cenacchi G., Tazzari P.L. (2021). An Abnormal Host/Microbiomes Signature of Plasma-Derived Extracellular Vesicles Is Associated to Polycythemia Vera. Front. Oncol..

[111] Shah R., Patel T., Freedman J.E. (2018). Circulating Extracellular Vesicles in Human Disease. N. Engl. J. Med..

[112] Elalaoui K., Weihe C., Oliver M.A., Craver M.B., Lai H.Y., Brooks S., Kim D., Martiny J., Whiteson K., Fleischman A. (2018). Investigating the Role of the Gut Microbiome in the Inflammatory State of Myeloproliferative Neoplasms. Blood.

[113] Oliver A., El Alaoui K., Haunschild C., Avelar-Barragan J., Luque L.F.M., Whiteson K., Fleischman A.G. (2022). Fecal Microbial Community Composition in Myeloproliferative Neoplasm Patients Is Associated with an Inflammatory State. Microbiol. Spectr..

[114] Pedersen K.M., Bak M., Sørensen A.L., Zwisler A.-D., Ellervik C., Larsen M.K., Hasselbalch H.C., Tolstrup J.S. (2018). Smoking is associated with increased risk of myeloproliferative neoplasms: A general population-based cohort study. Cancer Med..

[115] Leal A., Thompson C.A., Wang A.H., Vierkant R., Habermann T.M., Ross J.A., Mesa R.A., Virnig B.A., Cerhan J.R. (2013). Anthropometric, medical history and lifestyle risk factors for myeloproliferative neoplasms in The Iowa Women’s Health Study cohort. Int. J. Cancer.

[116] Iho S., Tanaka Y., Takauji R., Kobayashi C., Muramatsu I., Iwasaki H., Nakamura K., Sasaki Y., Nakao K., Takahashi T. (2003). Nicotine induces human neutrophils to produce IL-8 through the generation of peroxynitrite and subsequent activation of NF-κB. J. Leukoc. Biol..

[117] Pedersen K.M., Çolak Y., Bojesen S.E., Nordestgaard B.G. (2020). Low high-density lipoprotein and increased risk of several cancers: 2 population-based cohort studies including 116,728 individuals. J. Hematol. Oncol..

[118] Gleitz H.F., Benabid A., Schneider R.K. (2021). Still a burning question: The interplay between inflammation and fibrosis in myeloproliferative neoplasms. Curr. Opin. Hematol..

[119] Luque L.F.M., Blackmon A.L., Ramanathan G., Fleischman A.G. (2019). Key Role of Inflammation in Myeloproliferative Neoplasms: Instigator of Disease Initiation, Progression. and Symptoms. Curr. Hematol. Malign Rep..

[120] Rodríguez-García A., García-Vicente R., Morales M., Ortiz-Ruiz A., Martínez-López J., Linares M. (2020). Protein Carbonylation and Lipid Peroxidation in Hematological Malignancies. Antioxidants.

[121] Jang M., Kim S.S., Lee J. (2013). Cancer cell metabolism: Implications for therapeutic targets. Exp. Mol. Med..

[122] Musolino C., Allegra A., Saija A., Alonci A., Russo S., Spatari G., Penna G., Gerace D., Cristani M., David A. (2012). Changes in advanced oxidation protein products, advanced glycation end products, and s-nitrosylated proteins, in patients affected by polycythemia vera and essential thrombocythemia. Clin. Biochem..

[123] Durmus A., Mentese A., Yilmaz M., Sumer A., Akalin I., Topal C., Alver A. (2013). Increased oxidative stress in patients with essential thrombocythemia. Eur. Rev. Med. Pharmacol. Sci..

[124] Vener C., Novembrino C., Catena F.B., Fracchiolla N.S., Gianelli U., Savi F., Radaelli F., Fermo E., Cortelezzi A., Lonati S. (2010). Oxidative stress is increased in primary and post−polycythemia vera myelofibrosis. Exp. Hematol..

[125] Balaian E., Wobus M., Bornhäuser M., Chavakis T., Sockel K. (2021). Myelodysplastic Syndromes and Metabolism. Int. J. Mol. Sci..

[126] Kreitz J., Schönfeld C., Seibert M., Stolp V., Alshamleh I., Oellerich T., Steffen B., Schwalbe H., Schnütgen F., Kurrle N. (2019). Metabolic Plasticity of Acute Myeloid Leukemia. Cells.

[127] Méndez-Ferrer S., Bonnet D., Steensma D.P., Hasserjian R.P., Ghobrial I.M., Gribben J.G., Andreeff M., Krause D.S. (2020). Bone marrow niches in haematological malignancies. Nat. Rev. Cancer.

[128] Rashkovan M., Ferrando A. (2019). Metabolic dependencies and vulnerabilities in leukemia. Genes Dev..

[129] Reddy M.M., Fernandes M.S., Deshpande A., Weisberg E., Inguilizian H.V., Abdel-Wahab O., Kung A.L., Levine R.L., Griffin J.D., Sattler M. (2011). The JAK2V617F oncogene requires expression of inducible phosphofructokinase/fructose-bisphosphatase 3 for cell growth and increased metabolic activity. Leukemia.

[130] Rao T.N., Hansen N., Hilfiker J., Rai S., Majewska J.-M., Leković D., Gezer D., Andina N., Galli S., Cassel T. (2019). JAK2-mutant hematopoietic cells display metabolic alterations that can be targeted to treat myeloproliferative neoplasms. Blood.

[131] Gu Z., Liu Y., Cai F., Patrick M., Zmajkovic J., Cao H., Zhang Y., Tasdogan A., Chen M., Qi L. (2019). Loss of EZH2 Reprograms BCAA Metabolism to Drive Leukemic Transformation. Cancer Discov..

[132] Zhan H., Ciano K., Dong K., Zucker S. (2015). Targeting glutamine metabolism in myeloproliferative neoplasms. Blood Cells Mol. Dis..

[133] Gómez-Cebrián N., Rojas-Benedicto A., Albors-Vaquer A., Bellosillo B., Besses C., Martínez-López J., Pineda-Lucena A., Puchades-Carrasco L. (2021). Polycythemia Vera and Essential Thrombocythemia Patients Exhibit Unique Serum Metabolic Profiles Compared to Healthy Individuals and Secondary Thrombocytosis Patients. Cancers.

